# Effect of Pegbovigrastim on Hematological Profile of Simmental Dairy Cows during the Transition Period

**DOI:** 10.3390/ani9100841

**Published:** 2019-10-21

**Authors:** Francesca Trimboli, Valeria Maria Morittu, Antonio Di Loria, Andrea Minuti, Antonella Anna Spina, Fiorenzo Piccioli-Cappelli, Erminio Trevisi, Domenico Britti, Vincenzo Lopreiato

**Affiliations:** 1 Interdepartmental Services Centre of Veterinary for Human and Animal Health, Department of Health Science, Magna Græcia University, 88100 Catanzaro, Italy; trimboli@unicz.it (F.T.); morittu@unicz.it (V.M.M.); anto.spina90@gmail.com (A.A.S.); britti@unicz.it (D.B.); 2 Department of Veterinary Medicine and Animal Productions, University of Napoli Federico II, 80137 Napoli, Italy; 3 Department of Animal Sciences, Food and Nutrition, Faculty of Agriculture, Food and Environmental Science, Università Cattolica del Sacro Cuore, 29122 Piacenza, Italy; andrea.minuti@unicatt.it (A.M.); fiorenzo.piccioli@unicatt.it (F.P.-C.); erminio.trevisi@unicatt.it (E.T.); vincenzo.lopreiato@unicatt.it (V.L.)

**Keywords:** pegbovigrastim, simmental, transition period, hematology

## Abstract

**Simple Summary:**

During the transition period (TP), the innate and adaptive immune system of dairy cows is impaired, contributing to an increase in susceptibility to infectious disease. Pegbovigrastim is a recombinant form of the granulocyte colony-stimulating factor, that stimulates differentiation of hemopoietic stem cells to granulocyte and shortens maturation time within the bone marrow and their release in circulation. For the first time, this study investigated the effect of pegbovigrastim on the hematologic profile, including red cells and platelets during the TP in dual-purpose Simmental dairy cows. Results confirm the efficacy of pegbovigrastim administration in promoting the numbers of total with cells, mainly neutrophils. Nevertheless, this study highlighted the presence of an anemia condition in treated cows most likely caused by an inflammatory process involving the endothelium. Overall, this study confirms the safety of pegbovigrastim administration, but further studies need to check its ability to reduce infection even in Simmental breed.

**Abstract:**

Pegbovigrastim is a long-acting analog of recombinant bovine granulocyte colony-stimulating factor, that promotes and increases the count and functionality of polymorphonuclear cells in dairy cows. The present study aimed to explore, for the first time in Simmental cows, the clinical and hematological effect of pegbovigrastim during the transition period (TP). Cows were randomly assigned into two groups: treated group (PEG; n = 16) received pegbovigrastim at approximately 7 days before expected parturition and within 6 h after calving, and control group (CTR; n = 16) received saline solution. Blood samples were obtained at −7, 0, 1, 3, 7, 14, 21, and 30 days relative to calving. PEG group showed white blood cells (WBC) count consistently higher compared with CTR group (*p* < 0.001) until to 3 weeks after calving. Neutrophils remained higher in PEG group (*p* < 0.001) up to three weeks after calving, compared with CTR group, with slight increment of band cells. Moreover, PEG group displayed a lower index of myeloperoxidase at 1, 3, and 7 days after calving (*p* < 0.01) compared with CTR. Basophils and lymphocytes showed a similar trend to those observed for neutrophils at 1 day after calving in PEG group. Finally, monocytes remained markedly elevated until 3 days after calving in PEG compared to CTR group (*p* < 0.001), whereas in PEG group, eosinophils population showed lower percentage values at 1 and 3 days after calving but higher values at 30 days compared with CTR group. PEG group was characterized by lower red blood cells (RBCs) count compared with CTR group (*p* < 0.05) and higher % of red cell volume distribution width (RDW) from week 2 and mean corpuscular volume (MCV) at 30 days after calving. In addition, the mean platelet volume (MPV) was significantly higher in PEG group at calving, 1, 3, and 7 days after calving compared with CTR group (*p* < 0.05). For the first time, we described the effect of pegbovigrastim in a breed not specialized exclusively in milk production as Holstein, but with dual purpose (meat and milk), evaluating the complete hematological profile in cows during the transition period. These results provide evidence on the proliferative effect of pegbovigrastim on WBC in Simmental breed highlighting its possible side effect on RBCs.

## 1. Introduction

The transition period (TP) is one of the most critical physiological stages in dairy cattle, characterized by a negative energy balance and micronutrient deficiencies [1]. To satisfy the synthesis of colostrum and milk, high yielding cows need a higher demand of energy, but a decreased feed intake fails to let them adapt to this metabolic challenge. Immediately after parturition, dairy cows increase mobilization of body fat and subsequently serum NEFA levels [2], becoming more susceptible to several metabolic and infectious disorders affecting their productive and reproductive efficiency. An increased incidence of mastitis in cow was observed beginning 1 to 2 week before calving and continuing for several weeks after calving [3]. Moreover, during TP, an easier development of metritis was recorded in two different studies; the authors correlate this aspect with the impaired chemotaxis and activation of neutrophils [4] and with a reduction of neutrophils functions [5]. Although the exact mechanism for increase susceptible to pathogens infection is not fully clear yet, a suppression of immune responsiveness during the transition period in cow has been well documented [6]. Neutrophils represent the first defense line against microorganisms and play a crucial role of innate immune response; once at sites of infection, these cells actively phagocytize microorganisms or form neutrophil extracellular traps to kill pathogens [7]. Activated neutrophils release myeloperoxidase (MPO) to catalyze the synthesis of HOCl employed in bactericidal, fungicidal, and virucidal reactions. In recent years, it has become evident that neutrophils are also capable of modifying the overall immune response exchanging information with macrophages, dendritic cells, and other cells of the adaptive immune system through either soluble mediators or direct cell-cell contact [8]. Neutrophils are under the control of the granulocyte colony stimulating factor (G-CSF), whose effects on granulocytopoiesis are well known, and shown to have direct effects on the recruitment and bactericidal ability of neutrophils, resulting in improved survival of experimentally infected animals [9].

Since cows during TP are generally immunosuppressed and since bacterial infection is considered to stimulate granulocytopoiesis, in the last years recombinant G-CSF has become a therapeutic agent for preventive treatment of infections. In 2003, recombinant bovine G-CSF (bG-CSF) has been modified and the presence of a covalent binding of polyethylene glycol in PEGylated bovine G-CSF (pegbovigrastim) prolonged its half-life in the body while maintaining the induction of neutrophilia and increasing the phagocytic and cytotoxic activity of neutrophils [10,11,12]. Several studies showed that in cattle, the administration of pegbovigrastim provides a well-tolerated novel approach to overcoming periparturient immune suppression [13,14,15,16,17,18]. These studies, carried out mainly on Holstein and Jersey cows, demonstrated how the administration of pegbovigrastim results always able to increase count and function of circulating neutrophils while data on the effects on other leukocyte cells, as lymphocytes and monocytes are poor and contradictory. McDougall et al. [16] and Zinicola et al. [17] found an increament, whereas Van Schyndel et al. [19] observed a decrement of lymphocytes and monocytes count. In cattle, data on the effects of pegbovigrastim on red cells line are very poor, while no effects were described on platelets. McDougall [16] found a slight decrease of red cells count and hematocrit in cows treated at 7 and 21 days after calving [20]. In human hematological studies, side effects of G-CSF therapy include a slight decrease in hemoglobin concentration and in platelet count, although in rare cases, thrombocytopenia has been documented [21,22,23].

Thus, the aim of this study was to test pegbovigrastim for the first time in a dual-purpose Simmental breed during TP, in order to verify its effects on complete hematologic profile. The study conducted with a flow cytometry-based system, in addition to better define all cell blood population, explored myeloperoxidase index (MPXI), an independent and useful biomarker for the diagnosis and follow-up of infectious diseases.

## 2. Materials and Methods

The trial was carried out in accordance with Italian law on animal experimentation (DL n. 26, 04/03/2014) and received an institutional approval by Ethical Animal Care and Use Committee of the Magna Graecia University of Catanzaro (Protocol No. 302-5/5/2017). Trial started in November 2017 and ended in May 2018.

### 2.1. Animal Management and Treatment

Before the onset of the trial, a sample size calculation was calculated a priori: computation required sample size power analyses test (G*Power 3.1.9.2 for Windows, 2010–2018 Heinrich-Heine-Universität Düsseldorf). The neutrophil populations outcomes were used to calculate the samples size power. Data of herd hematological profile of the farm, where trial was performed (not published), were employed to calculate standard deviation and effect size. Standard deviation of herd neutrophil population (all cows sampled) was 1.45 × 10^3^/µL and value of effect size f was 2.29. Based on sample size power test, a minimum number of 14 cows for each group was required to ensure a power (1–β err prob) of 0.80. Based on investigators criteria, cows were eligible for inclusion if healthy after clinical examination, did not show mastitis before drying off in the previous lactation, had a parturition with a single calf, WBC and neutrophil count were in normal range and not received any antimicrobial treatment within 15 days prior to inclusion in the study. The commercial farm (Fattoria Demetra, Calabria, Italy), eligible for the purpose of this study, consisted of modern buildings, with 60 lactating cows loose housed and fed using the total mixed ration (TMR) technique. The average of milk production of the farm at the beginning of the study was 27.43 kg/day with an average of days in milk of 175 days. On average, fat content was 3.87% and protein content 3.46%. Thus, 36 Simmental cows under a dairy management met the inclusion criteria and were enrolled in the trial. These 36 cows were blocked by calving date, body condition score (scale 1 to 5), and parity. Within each block, cows were randomly assigned (by flipping a coin) into 2 groups: treated group (PEG; 18 cows) received pegylated recombinant bovine granulocyte colony stimulating factor (rbG-CSF; pegbovigrastim; Imrestor; Elanco Animal Health), and control group (CTR; 18 cows) received saline solution. Treatments were administrated via subcutaneous injection in the scapular region using prefilled syringes, provided by the manufacturer (Elanco Animal Health, Liverpool, UK) for PEG group (15 mg of pegbovigrastim in 2.7 mL solution) and syringes with 18-gauge × 2.5-cm needle for CTR group (2.7 mL of sterile saline). The first dose was administrated approximately 7 days before expected parturition, depending on judgment of investigators based on physical changes including swelling of vulva and filling of udder. The second dose was administrated within 6 h after calving. Cows were excluded from the study if developed any disease after inclusion, died, left the herd for any reason, or received any therapeutic treatment. From the animals initially selected (n = 36), 4 were excluded: 2 cows because the interval between the first injection of pegbovigrastim and parturition was not respected, one cow because developed severe milk fever and one because had twin calving. The remained 32 Simmental cows (PEG = 16 cows; CTR = 16 cows) involved for the final evaluation herein did not suffer from any acute health disorders during the entire experimental period. The average of parity was 2.44 ± 1.71 for PEG cows and 2.20 ± 1.33 for CTR cows (*p* = 0.23). Investigators involved in allocation of animals to treatment groups, administration of treatments, and blood samples were different from operators involved in the animal management and also from operators involved in laboratory analysis. Thus, farm operators, the veterinary responsible for clinical and reproductive inspections, and operators in the laboratory for blood analysis were blinded to treatment assignments. Each blood sample was labeled with a unique code number that only investigators in the field and not in the laboratory knew. Dry cows were housed in a free walking straw barn, and after parturition cows were moved to lactation barn still free walking straw system. According to the farm management, cows were fed once daily (0700 and 0800 h, lactating and dry cows respectively) with TMR technique, and after parturition they were milked twice daily (at 0500 and 1700 h). Diets composition used in close-up and early lactation period are reported in Table 1. At the end of the trial, cows enrolled in the study proceeded the productive career under the management procedure of the farm.

### 2.2. Blood Sample Collection and Complete Blood Count Analysis

Blood samples, before TMR delivery, were collected in the morning from the jugular vein at −7 (before first pegbovigrastim injection), 0 (before second pegbovigrastim injection), 1, 3, 7, 14, 21, and 30 days relative to parturition. Blood samples were collected using an 18-gauge Vacutest Kima needle (Vacutest Kima srl, Arzergrande, Italy) into 9-mL blood collection tubes (Vacuette, Austria) containing K3EDTA and stored at + 4 °C. Samples were analyzed within 2 h of collection using an ADVIA 2120 Hematology System machine (Siemens, Germany).

The parameters taken into account in the current study were total white blood cell (WBC) count, WBC differential count for neutrophils, eosinophils, basophils, lymphocytes, and monocytes as percentage and absolute number, red blood cells count (RBC), hematocrit value (HCT), hemoglobin concentration (HGB), mean corpuscular volume (MCV), mean corpuscular hemoglobin (MCH), hemoglobin concentration distribution width (HDW), RBC distribution width (RDW). In addition, platelet indices were also analyzed and included platelet count (PLT), mean platelet volume (MPV), PLT volume distribution width (PDW), plateletcrit (PCT), and MPXI.

MPXI is calculated as MPXI = [(Mean Neutrophil Region MPO-Expected Staining Index)/Expected Staining Index] × 100, where the Mean Neutrophil Region MPO is the result of the absorbance measurement in the neutrophil region. The Expected Staining Index is the expected MPO measurement result for an ideal standard neutrophil population; it is a technical constant and is maintained by regular calibration. A negative MPXI value means that the sample cells contain less peroxidase than the ideal normal population; a positive value means higher MPO content compared to the ideal normal population.

### 2.3. Milk Collection, Milk Yield, and Analysis

Milk samples were collected at 1, 3, 7, 14, 21, and 30 days in lactation and milk production was recorded in the same time points from consecutive morning and evening milking. Milk samples were analyzed separately for fat, protein, lactose, casein, and urea by mid-infrared procedures using the Milkoscan FT + (Foss Electric A/S, Hillerød, Denmark).

### 2.4. Statistical Analysis

Hematology data were subjected to ANOVA and analyzed using repeated measure statement by MIXED procedure of SAS version 9.4 (SAS Institute Inc., Cary, NC, USA) according to the following model:

*Y_ijkl_* = *μ* + *G_i_* + *T_j_* + *GT_ij_* + *c_m:ij_* + *ε_ij;_* (1)

where *Y_ijkl_* = dependent continuous variable, *μ* = over-all mean, *G_i_* = fixed effect of treatment (*i* = PEG vs. CTR), *T_j_* = fixed effect of time (days; −7, 1, 3, 7, 14, 21, and 30), *GT_ij_* = interaction between treatment and time; *c_m:ij_* = random effect of *m*th animal (cow) nested within treatment; and *ε_ij_* = residual error. The Kenward-Roger statement was used for computing the denominator degrees of freedom, whereas spatial power was used as the covariance structure. Normality of data was checked by Univariate procedure of SAS (ver. 9.4). Variables not normal distributed were log10 transformed and once the output data carried out from analysis, they were back-transformed. The significance was declared at a *p* ≤ 0.05 whereas, tendency was declared at 0.10 ≥ *p* > 0.05 using the PDIFF statement in SAS (ver. 9.4).

## 3. Results

### 3.1. Milk Production and Quality

The average of milk production during the first month of lactation (Table 2) was not different between PEG and CTR group (Trt, *p* = 0.77; Trt × Day, *p* = 0.39). In addition, no differences were observed for milk quality traits (Trt, *p* > 0.10; Trt × Day, *p* > 0.10).

### 3.2. Effect of Pegbovigrastim on Total WBC Count and on Differential WBC Count

**Figure 1:**
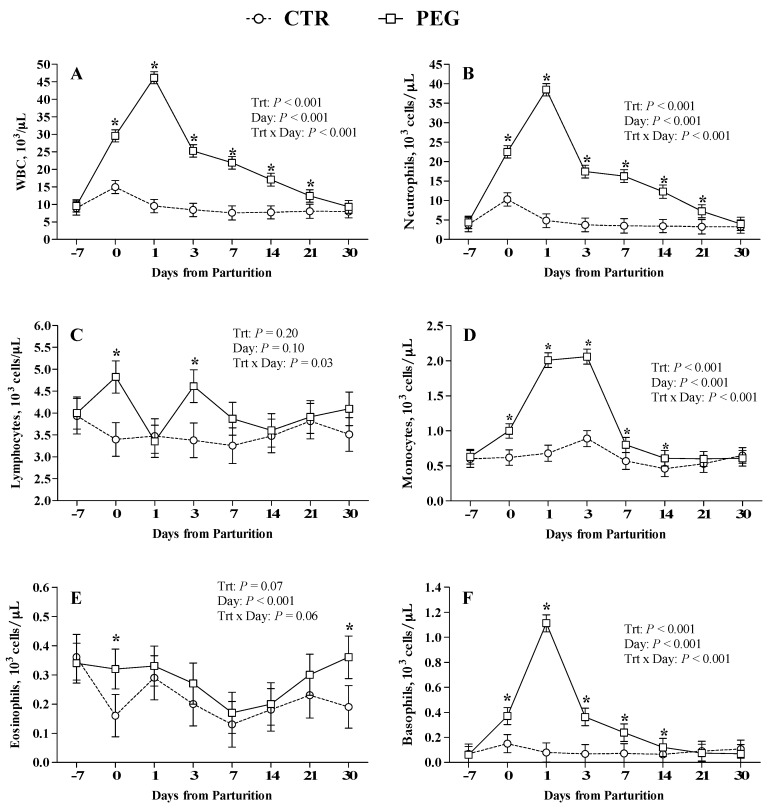
Effect of Pegbovigrastim on total white blood cell (WBC) count and differential WBC count. Least-squares means ± standard error of (**A**) circulating total whole blood leukocytes (WBC × 10^3^/μL), (**B**) neutrophils (×10^3^/μL), (**C**) lymphocytes (×10^3^/μL), (**D**) monocytes (×10^3^/μL), (**E**) basophils (×10^3^/μL) and (**F**) eosinophils count (×10^3^/μL) during the transition period of Simmental cows treated either with 2 subcutaneous injections of pegbovigrastim (PEG) or saline solution (CTR) at −7 days relative to calving and within 6 h after calving. Asterisks (*) indicate differences at *p* < 0.05.

**Figure 2:**
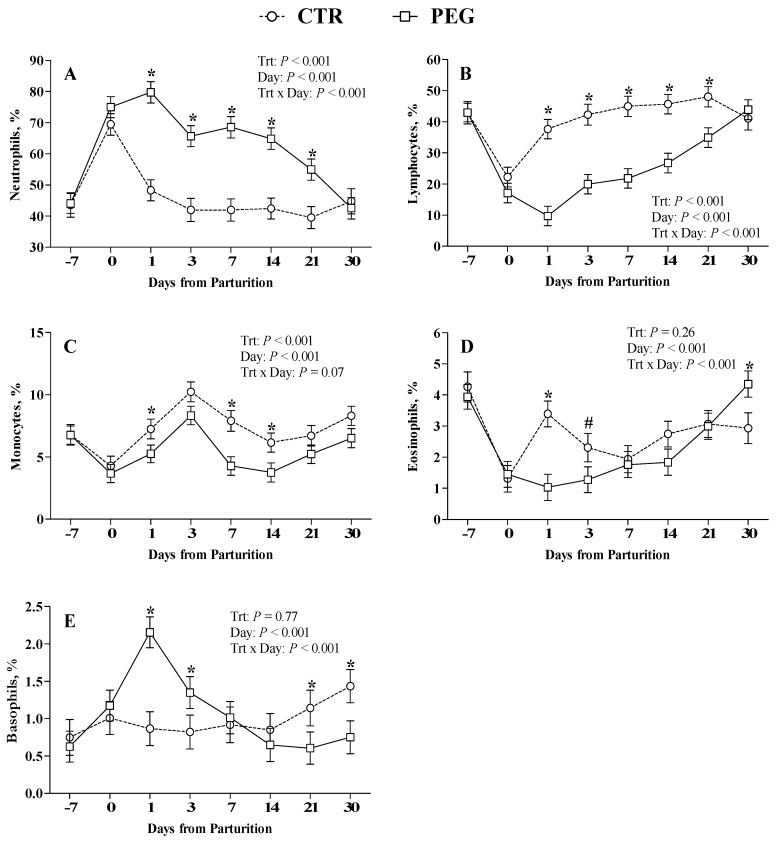
Effect of Pegbovigrastim on differential WBC count. Least-squares means ± standard error of neutrophils (**A**), lymphocytes (**B**), monocytes (**C**), basophils (**D**), and eosinophils (**E**) percentage during the transition period of Simmental cows treated either with 2 subcutaneous injections of pegbovigrastim (PEG) or saline solution (CTR) at −7 days relative to calving and within 6 h after calving. Asterisks (*) indicate differences at *p* < 0.05, whereas hashtags (#) indicate tendency at 0.10 ≥ *p* > 0.05.

All blood samples were analyzed for total WBC count and differential cell count was carried out as in percentage (%) and as in absolute values (n° cell × 10^3^/µL). Excluding eosinophil (% and n° cell × 10^3^/µL) and lymphocyte percentage, all other cell populations were not normally distributed, thus they were log10 transformed for statistical analysis. For ease of interpretation, normalized last square means and standard errors were properly back-transformed. There were significant interactions between treatment and time for WBC count (*p* < 0.001; Figure 1A), percentage and absolute value of neutrophils (Figure 1B and Figure 2A, respectively) and basophils (*p* < 0.001; Figure 1F and Figure 2E, respectively), percentage of lymphocytes and eosinophils (*p* < 0.001; Figure 2B,D, respectively), and absolute value of monocytes (*p* < 0.001; Figure 1D). The WBC concentration in the PEG group increased strongly at parturition (6 days after the first dose of pegbovigrastim) and further increased during the 24 h after the injection of second dose (Figure 1A). Then, it gradually decreased during the three weeks after calving. During this period the WBC count in PEG group was consistently higher compared with CTR group (*p* < 0.001) until to 3 weeks after calving. CTR group showed a slight increase in WBC count at calving, which decreased the following day.

**Figure 3:**
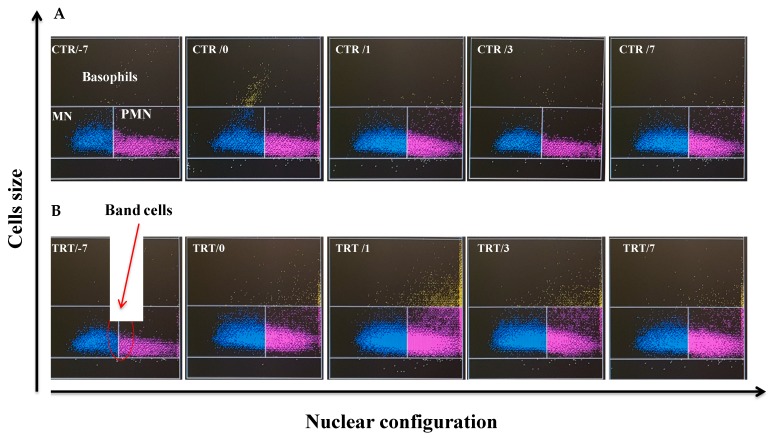
Effect of Pegbovigrastim on band cells evaluating baso cytogram provided by ADVIA 2120 Hematology Analyzer. Representative cytograms during the transition period of Simmental cows treated either with saline solution (CTR; **A**) or 2 subcutaneous injections of pegbovigrastim (PEG; **B**) or at −7 days relative to calving and within 6 h after calving. In blue are indicate mononuclear (MN) while in red polymorphonuclear (PMN) populations: band cells (red circle) appear between the MN and PMN populations.

Analyzing in detail the leukocyte populations, the neutrophils count trend reflects the WBC pattern with a marked increase of its percentage at parturition compared with the week before, and again at 24 h from the second dose (calving time). In PEG group, the percentage of neutrophils was higher (*p* < 0.001) at 1 day from calving compared with CTR group (Figure 2A), whereas their number at parturition was higher (*p* < 0.001) compared with CTR cows (Figure 1B). Neutrophils (% and absolute value) remained higher in PEG group (*p* < 0.001) up to three weeks after calving, compared with CTR group. The examination of CTR and PEG groups Baso cytograms (Figure 3) pointed out a slight, but clear increment of band cells.

**Figure 4:**
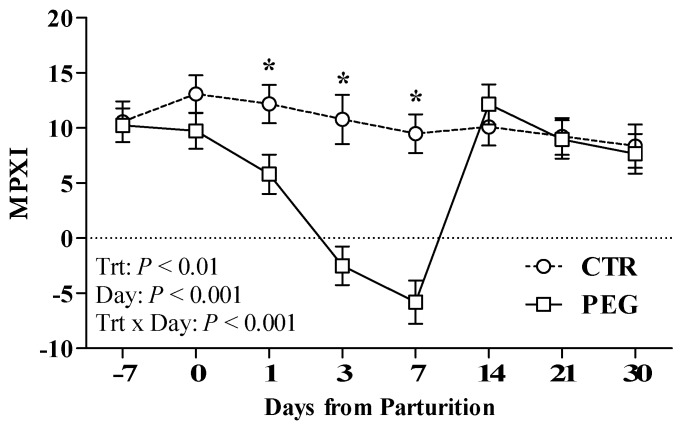
Effect of Pegbovigrastim on MPXI. Least-squares means ± standard error of neutrophil myeloperoxidase index (MPXI) are evaluated by using ADVIA 2120 Hematology Analyzer during the transition period of Simmental cows treated either with 2 subcutaneous injections of pegbovigrastim (PEG) or saline solution (CTR) at −7 days relative to calving and within 6 h after calving. Asterisks (*) indicate differences at *p* < 0.05.

MPXI values rapidly decreased in PEG group after pegbovigrastim administration, reaching negative values at 3 and 7 days after calving and returning to normal values at 14 days (Figure 4). In addition, PEG group displayed lower MPXI values at 1, 3 and 7 days after calving compared with CTR cows (*p* < 0.01; Figure 4).

The basophil population (absolute values) showed a similar trend to those observed for neutrophils with a peak at 1 day after calving in PEG group (Figure 1F). The % of basophils in PEG group reached the highest value at 1 day after calving and then returned to level of CTR group after 7 days (Figure 2E).

The administration of pegbovigrastim led to a significant increase in percentage of lymphocytes only after the second dose compared with CTR group in the same period (Figure 2B). The % of lymphocytes in PEG group remained higher compared with CTR group during the following three weeks. The % of lymphocytes in CTR group tended to increase reaching PEG group levels only 30 days after parturition.

Pegbovigrastim treatment induced a negative effect on eosinophils population. Compared with CTR group, PEG group showed lower percentage values at 1 and 3 days after calving but higher values at 30 days. However, CTR group had showed a marked increase at 1 and 3 after calving compared with the calving time (Figure 2D).

The effects of pegbovigrastim on monocytes number was significant at the time of calving and monocytes remained markedly elevated until 3 days after calving compared with CTR group (Figure 1D). Pegbovigrastim did not affect lymphocytes number, eosinophils number, and monocytes percentage, with similar values between PEG and CTR groups (Figure 1C,E, Figure 2C, respectively).

### 3.3. Effect of Pegbovigrastim on RBCs, Platelets Count, and Their Parameters

**Figure 5:**
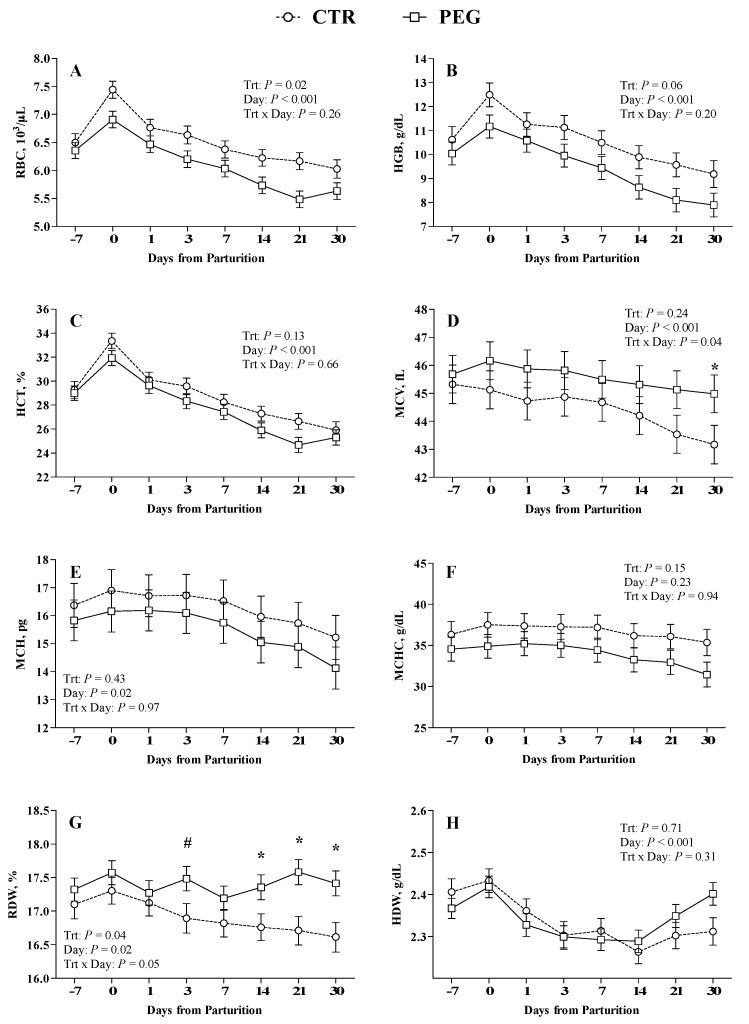
Effect of Pegbovigrastim on red blood cells (RBCs) and their parameters. Least-squares means ± standard error of (**A**) red blood cell count (RBC, ×10^6^/μL) (**B**) hemoglobin (HGB, g/dL), (**C**) hematocrit (HCT, %), (**D**) mean corpuscular volume (MCV, fL), (**E**) mean corpuscular hemoglobin (MCH, pg), (**F**) mean corpuscular hemoglobin concentration (MCHC, g/dL), (**G**) red cell volume distribution width (RDW, %), and (**H**) hemoglobin concentration distribution width (HDW, g/dL) during the transition period of Simmental. Asterisks (*) indicate differences at *p* < 0.05, whereas hashtags (#) indicate tendency at 0.10 ≥ *p* > 0.05.

Overall, PEG group was characterized by lower RBCs count compared with CTR group (*p* < 0.05; Figure 5A). The administration of pegbovigrastim did not show any effect on HGB and HCT concentration (*p* > 0.05; Figure 5B,C, respectively). No differences were obtained between PEG and CTR group for MCH, MCHC and HDW (Figure 5E,F,H, respectively). In the PEG group, the % of RDW increased significantly from 2th week after calving (Figure 5G). Moreover, MCV in PEG group was significantly higher compared with CTR group at 30 days after calving (Figure 5D). Normocytic hypochromic anemia was detected in 3/16 animals (19%) of CTR group and in 8/16 animals (50%) of PEG group based on reference interval of hematological parameters (HGB: 8.4–12 g/dL; MCV: 36–50 fL; MCHC: 38–43 g/dL).

**Figure 6:**
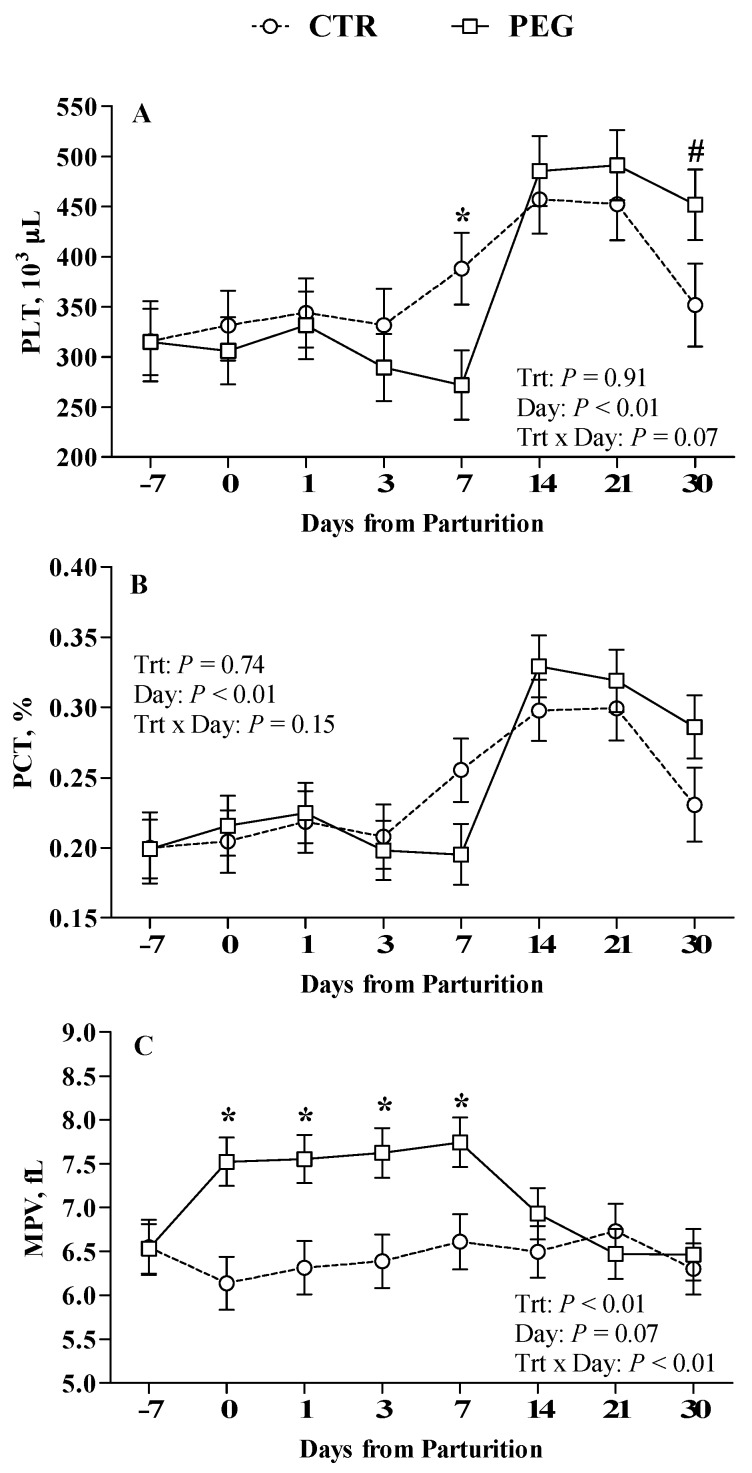
Effect of Pegbovigrastim on PTLs and their parameters. Least-squares means ± standard error of (**A**) platelet count (PLT, ×10^3^/μL), (**B**) platelet crit (PCT, %), and (**C**) mean platelet volume (MPV, fL) during the transition period of Simmental cows treated either with 2 subcutaneous injections of pegbovigrastim (PEG) or saline solution (CTR) at –7 days relative to calving and within 6 h after calving. Asterisks (*) indicate differences at *p* < 0.05, whereas hashtags (#) indicate tendency at 0.10 ≥ *p* > 0.05.

Regarding PLT and PCT, no differences were observed between PEG and CTR groups (Figure 6A,B, respectively), whereas MPV was significantly higher in PEG group at calving and at 1, 3, and 7 days after calving compared with CTR group (Figure 6C; *p* < 0.05). In 14/16 animals (87.5%) of PEG group and in 6/16 animals (37.5%) of CTR group MPV value was higher than normal range (4.6–7.4 fL).

## 4. Discussion

### 4.1. Effect of Pegbovigrastim on Total WBC Count and on Differential WBC Count

In almost all studies previously published, the effect of pegbovigrastim on hematological parameters of periparturient cows mainly concerned Holstein and Jersey cows [15,16,17,19], with the exception of one study conducted on Lithuanian Black and White cows [25]. Most of these studies were focused especially on circulating neutrophils, the main leukocyte population influenced by pegbovigrastim and partly on lymphocytes and monocytes [15,16,17,19]. A slight decrease of red cells count and hematocrit in cows treated with pegbovigrastim were reported only by McDougall [16]. No data are available on the effects of pegbovigrastim administration on platelets. At our knowledge, this is the first report focusing on influences of pegbovigrastim on complete blood cell population of periparturient Simmental cows.

According to this function, in Simmental cow the administration of pegbovigrastim at approximately 1 week before calving and within 6 h after calving triggered a broad increment in circulating total white cells that remained upper to normal range up to 21 days after last injection. While in CTR group WBC count reached the highest peak the day of partum, in PEG group this maximum value was obtained the day after calving establishing a state of neutrophilia (up to 80% of leukocytes were neutrophils). The trend of WBCs and neutrophils observed following pegbovigrastim administration is consistent with previous data reported by other authors [13,15,16,17,19]. Moreover, according to results obtained from Holstein and Jersey cows [16,17] and calves [20], we observed a slow decrease of neutrophil count in PEG group indicative of prolonged activity of pegbovigrastim. According to other authors [15,19,26], neutrophilia induced by pegbovigratim was characterized by a ‘left-shift’ towards progenitor cells with a release of mature neutrophils and band cells from storage pool in bone marrow. The rbG-CSF besides is effect on the numerical increase of neutrophils is also able to influence their activities. The reduced MPXI values recorded in PEG group could be in fact indicative of a marked neutrophil degranulation and MPO release. In severe bacterial infections, such as sepsis, a natural stimulation of production of endogenous granulocyte colony-stimulating factor is able to reduce MPXI values, releasing large amounts of MPO for bactericidal activities from activated neutrophils [27]. In our study condition, the administration of pegbovigrastim seems to replicate this aspect showed by a decreased amount of MPXI values. This effect appeared strictly associated to the dose administrated, becoming reversible within 14 days of the treatment, when MPXI values appeared normalized with those of CTR group.

The incremented release of MPO, along with the increased number of circulating neutrophils ready to go to the site of infection observed after rbG-CSF treatment, could improve the ability of cows to prevent clinical disease during periparturient period.

In PEG group, the increments detected on monocytes populations are in line with studies conducted in cows by McDougall et al. [16] and Zinicola et al. [17] and in calves by Kegles et al. [20]. Our results showed a substantial transitory increase of circulating monocytes absolute values, not affecting the percentage. On human monocytes, the presence of G-CSF receptors suggests a regulation of this cell population via G-CSF [28], however this hypothesis must be verified in cattle.

Although circulating lymphocytes number was found positively influenced by rbG-CSF in Holstein, Jersey, and Lithuanian Black and White cows [16,17,25], in our study condition no significant differences were observe between PEG and CTR groups. On the other hand, the percentage of lymphocytes in PEG group is clearly lower than CTR group, reasonable with the strong increment of percentage of neutrophils after rbG-CSF administration that became the predominant leukocyte population. The lack of effects of rbG-CSF on lymphocytes count is not surprising since lymphocytes and neutrophils derived from different progenitors and therefore lymphocytes should not be influenced by treatment with rbG-CSF.

Little is known about effects of rbG-CSF on basophils and eosinophils populations. Although rbG-CSF is a growth factor specific for neutrophil, we observed a strong increment of basophils count in PEG group, which resulted in contrast with data reported by van Schyndel [19] in Holstein cows. The implication of basophils increment in PEG group is not clear and further studies are needed to elucidate the meaning of their increase.

Finally, we did not find differences in eosinophils count in PEG group respect to CTR group differently by reported by van Schyndel et al. [19] that observed a slight decrease of eosinophils the day after the administration of second dose of rbG-CSF.

### 4.2. Effect of Pegbovigrastim on Rbcs, Platelets Count, and Their Parameters

Administration of pegbovigrastim resulted in a lower RBCs count compared with CTR group while did not show any effect on HCT and HGB. These results are partially in accord with McDougall et al. [16] that observed both lower count of RBC and a low value of HCT.

Although the values remained within the reference ranges, in PEG group, the increased % of RDW at 2 weeks after calving could be related to the general effect of pegbovigrastim on bone marrow function. Moreover, MCV in PEG group is significantly higher compared with CTR group at 30 days after calving. The finding of a normocytic, hypochromic anemia observed in 50% (8/16) and 19% (3/16) of the animals of the PEG and CTR groups, respectively, could show a boost anemic effect of the treatment [29]. Although this form of anemia is associated with iron deficiencies, this form can certainly be linked to nutrient deficiency typical of TP of cow [30].

The reason and the significance of lower concentration of RBC in PEG group than CTR group is not clear and further studies are necessary to elucidate these aspects.

In an experimental mouse model, Chang et al. [31] found that G-CSF is able to influence erythropoiesis by promoting newly synthesized erythrocytes faster than erythropoietin.

In humans a slight but significant decrease in hemoglobin concentration and platelet count has been observed as hematological effect of G-CSF treatment [32,33,34]. In cattle conversely, our data did not show any significant modification of platelets and their associated parameters except for MPV, after rbG-CSF treatment. In 14/16 animals exceeded the higher range of MPV indicating how PEG administration is able to increase of platelets volume. These data are in according to Ihara et al. [35] that reported the same trend in human after G-CSF administration. This effect is probably related to an inflammatory process and activation of endothelial cells, induced by G-CSF treatment. Large platelets are more active enzymatically and metabolically than small platelets and produce, for example, more thromboxane A2 [36].

## 5. Conclusions

This is the first study that exhaustively highlights the effects of pegbovigrastim on erythrocytes and platelets in Simmental cows. Specifically, we observed the presence of an anemia condition in the treated cows with which an increase in MPV was most likely caused by an inflammatory process involving the endothelium. These results represent a possible side effect of rbG-CSF administration and it should be carefully evaluated in future in order to fully understand the underlying mechanism and its impact on cow health status.

We observed in pegbovigrastim treated Simmental cows the same trend described in literature for neutrophils, monocytes, and basophils number, the last not previously reported. Moreover, we describe for the first time the effects of pegbovigrastim on MPXI that markedly decrease: this observation could point to a major bactericidal activity of neutrophils in cows treated confirming its beneficial effects.

Overall data obtained in this study confirm the safety of pegbovigrastim administration, but further studies needed for check its ability to reduce infection even in Simmental breed.

## Figures and Tables

**Table 1:** Ingredient and nutrient composition of close-up (from −21 to parturition) and early lactation (from parturition to 30 DIM) diets for Simmental dairy cows treated with pegbovigrastim (PEG) or with saline (CTR) at approximately d −7 relative to calving and on the day of calving within 6 h.

Item	Close-Up	Lactation
Ingredient, % of DM		
Alfalfa hay, second or later cuts	-	25.00
Grass hay	73.50	24.42
Corn grain, ground, dry	11.78	27.12
Soybean meal	-	-
Commercial concentrate ^1^	14.71	22.04
Minerals and vitamins	-	1.42
Nutrient composition, % of DM		
CP	11.16	15.05
Starch	11.12	23.09
Ether extract	2.22	2.80
NDF	49.08	36.87
Forage NDF	44.11	28.33
ADF	25.40	21.94
ADL	3.74	4.29
NE_L_,^2^ Mcal/kg of DM	1.46	1.63

^1^ Conteined: flour extraction of toasted soybean, flour extraction of sunflower, dried stillage corn, wheat bran, alfalfa flour, middlings wheat, calcium carbonate, carob flour, fatty acid salts of palm oil, sodium chloride, molasses, phosphate dicalcium, sodium bicarbonate. ^2^ According to NRC [24] and calculated using Razio-Best software of Università Cattolica del Sacro Cuore (Piacenza, Italy).

**Table 2:** Effect of Pegbovigrastim injections to Simmental dairy cows during the fresh period (from 1 to 30 days in lactation) on milk yield and quality.

Item	PEG	CTR	SEM ^1^	*p*-Value		
				Trt	Day	Trt × Day
Milk yield, kg/day	23.62	24.08	1.16	0.77	<0.01	0.39
Fat, %	3.70	3.74	0.14	0.84	<0.01	0.86
Protein, %	3.32	3.35	0.05	0.61	<0.01	0.61
Casein, %	2.55	2.56	0.04	0.89	<0.01	0.75
Lactose, %	4.83	4.77	0.04	0.30	<0.01	0.24
Urea, mg/dL	25.95	27.12	1.02	0.42	<0.01	0.40

^1^ Greatest SEM.
